# Global analysis of the differentially expressed miRNAs of prostate cancer in Chinese patients

**DOI:** 10.1186/1471-2164-14-757

**Published:** 2013-11-05

**Authors:** Hui-chan He, Zhao-dong Han, Qi-shan Dai, Xiao-hui Ling, Xin Fu, Zhuo-yuan Lin, Ye-han Deng, Guo-qiang Qin, Chao Cai, Jia-hong Chen, Fu-neng Jiang, Xingyin Liu, Wei-de Zhong

**Affiliations:** 1 Department of Urology, Guangdong Key Laboratory of Clinical Molecular Medicine and Diagnostics, Guangzhou First People’s Hospital, Guangzhou Medical University, Guangzhou 510180, China; 2 Guangdong Provincial Institute of Nephrology, Southern Medical University, Guangzhou 510515, China; 3 Urology Key Laboratory of Guangdong Province, Guangzhou Medical University, Guangzhou 510230, China; 4 Department of Genetics, Albert Einstein College of Medicine, Bronx, New York 10461, USA

**Keywords:** Prostate cancer, miRNA expression profile, miR-374b, mRNA expression profile

## Abstract

**Background:**

Our recent study showed the global physiological function of the differentially expressed genes of prostate cancer in Chinese patients was different from that of other non-Chinese populations. microRNA are estimated to regulate the expression of greater than 60% of all protein-coding genes. To further investigate the global association between the transcript abundance of miRNAs and their target mRNAs in Chinese patients, we used microRNA microarray approach combined with bioinformatics and clinical-pathological assay to investigate the miRNA profile and evaluate the potential of miRNAs as diagnostic and prognostic markers in Chinese patients.

**Results:**

A total of 28 miRNAs (fold change ≥1.5; *P* ≤ 0.05) were differentially expressed between tumor tissue and adjacent benign tissue of 4 prostate cancer patients.10 top Differentially expressed miRNAs were validated by qRT-PCR using all 20 tissue pairs. Compared to the miRNA profile of non-Chinese populations, the current study showed that miR-23b, miR-220, miR-221, miR-222, and miR-205 maybe common critical therapeutic targets in different populations. The integrated analysis for mRNA microarray and miRNA microarray showed the effects of specifically inhibiting and/or enhancing the function of miRNAs on the gene transcription level. The current studies also identified 15 specific expressed miRNAs in Chinese patients. The clinical feature statistics revealed that miR-374b and miR-19a have significant correlations with clinical-pathological features in Chinese patients.

**Conclusions:**

Our findings showed Chinese prostate cancer patients have a common and specific miRNA expression profile compared with non-Chinese populations. The miR-374b is down-regulated in prostate cancer tissue, and it can be identified as an independent predictor of biochemical recurrence-free survival.

## Background

MicroRNAs (miRNAs) are small non-coding RNA molecules of 21-23nt that have deregulated expression in human cancer tissue [1]. Their deregulation has been shown to play a vital role in cancer initiation, progression and metastasis [2] and miRNAs represent a promising new class of cancer biomarker [3,4]. Recent studies demonstrate that miRNA expression patterns serve as phenotypic signatures of different cancers and could be used as diagnostic, prognostic and therapeutic tools [5]. Prostate cancer is the most common cancer in men in western countries [6]. A few studies have analyzed global microRNA expression profiles or the functional role of microRNAs in prostate cancer [7,8]. However the results showed highly inconsistent [8].

Prostate cancer occurrence in the Chinese populations was lower than that of the western countries. Our recent study showed the global physiological function of the differentially expressed genes of prostate cancer in Chinese patients was different from that of other non-Chinese populations [9], which suggest that the deregulation of cell cycle progression maybe a “key” to differentiate the epidemiological differences between Chinese and non-Chinese. On the one hand, miRNAs are estimated to regulate the expression of greater than 60% of all protein-coding genes [10]. On the other hand, accumulating evidence suggests that miRNAs can contribute to tumorigenesis either by directly modulating oncogenic or tumor suppressor pathways [4]. So it should be kept in mind that there are genetic characteristics of prostate cancer patients from different populations. In the last few years, increasing evidence has documented the role of microRNAs as new broad-spectrum oncogenes or tumor suppressor genes, thus their use as diagnostic, prognostic and therapeutic biomolecules is envisaged [11]. So more investigation on the expression profiles of miRNAs is needed to understand of the role of miRNAs in the development and progression of prostate cancer in different populations.

Recent studies have shown that microRNA inhibitory activity can be quantified by examining their target mRNA expression levels [12]. Therefore, in order to functionally investigated differentially expressed miRNAs targets, all of patients samples in this study are same as previous report about mRNA transcript study [9]. The objective of this study was to evaluate the potential of miRNAs as diagnostic and/or prognostic markers and to further provide a solid data basis for further functional analyses of prostate cancer-relevant miRNAs in Chinese patients.

## Method

### Patients and sample collection

Prostate tissues were obtained from the tissue bank at Guangzhou Medical University. All patients who participated in the study had given informed consent. The collection of the tissue specimens in accordance with the protocol was approved by the Institutional Ethical Board of Guangzhou Medical University. The clinic pathological data of these patients are summarized in Additional file 1: Table S1. The frozen tissues were procured immediately after surgery, cut into the size of ~1 cm^2^ × 0.5 cm pieces, fast-frozen in liquid nitrogen and stored at -80°C.

### Sample LCM and RNA preparation

The whole sample treatment and RNA extraction process were previously described by Chen JH et al. [9].

### miRNA expression microarray

Microarray experiments were carried out using 1-color hybridizations on human miRNA Microarray (V3, 8x15K) (Agilent, Cat No.:G4471A-021827), One glass slide formatted with eight high-definition 15 K arrays, based on Sanger miRbase (release 12.0), Probes: 866 human and 89 human viral miRNAs represented. The miRNA expressions data are available at GEO (http://www.ncbi.nlm.nih.gov/geo/query/acc.cgi?acc=GSE34932), which included a total of 16 samples data.

### Microarray data analysis

The microarray image information was converted into spot intensity values using Scanner Control Software Rev. 7.0 (Agilent). The signal after background subtraction was exported directly into the GeneSpring GX11 software (Agilent) for normalization. The mean normalized signal from biological replicates was used for comparative expression analysis. Paired t-test with Benjamini-Hochberg correction (P value ≤ 0.05) was used to identify differentially expressed miRNAs between tumor and benign tissues. The fold changes of expression signals between tumor and benign samples were calculated from the normalized values. Hierarchical clustering was performed with Pearson correlation using the differentially expressed miRNAs. 4 pairs cancer and benign tissues data (C2,C3,C4,C5,N2,N3,N4,N5) were used for further analysis.

### Confirmation of miRNA expression

For miRNA detection, qRT-PCR was processing as the protocol of All-in-One™ miRNA qRT-PCR Detection Kit (Cat No.:AOMD-Q020, GeneCopoeia, China) described. All assays were carried out in triplicate. miRNA expression in each sample was normalized with the house keeping gene (RNU6B and hsa-miR-130b) expression. The specificity of amplification was confirmed by melting curve analysis and also by running PCR products on agarose gels (3%). Relative quantification of target miRNA expression was evaluated using the comparative cycle threshold (CT) method.

MiRCURY LNA™ Detection probes 5′-DIG labeled (Cat No.:18091–01 for miR-19a, 38748–01 for miR-374b, 99002-01 for U6, Exiqon, Denmark) were used to detect the in situ hybridization signal for target miRNA on formalin-fixed or frozen section of prostate tissues. In situ hybridization was performed according to the manufacturer’s instructions of Enhanced sensitivity of in situ hybridization detection kit (peroxidase) (Cat No.: MK1030, Boster, China). The result scoring of in situ hybridization experiment was detail described by He HC et al. [13].

### Target gene prediction and integrated analysis by IPA

The selected miRNAs were further analyzed to identify the networks and pathways targets. Target prediction using TargetScan, combined with bioinformatics analysis (Ingenuity Pathway Analysis, IPA). The identified targets were compared with mRNA microarray (GEO Accession No: GSE28204) from the same patients to evaluate the implications of the altered miRNA expression [9].

### Statistical analysis

Data comparing differences in levels of expression of miRNAs between tumor tissue and adjacent benign tissue were analyzed using paired Student’s t-test by the software of Genespring version GX 11 (Agilent Inc, USA). Other data were statistically analyzed by the software of SPSS version13.0 for Windows (SPSS Inc, USA). Continuous variables were expressed as $\bar{X}\pm s$, paired Student’s t-test for qRT-PCR data, independent t-test for in situ hybridization result, Kaplan-Meier method for biochemical recurrence free-survival, and Cox regression model for the univariate and multivariate analysis. Differences were considered statistically significant when P < 0.05.

## Results

### miRNA expression profiling of clinical samples

**Figure 1:**
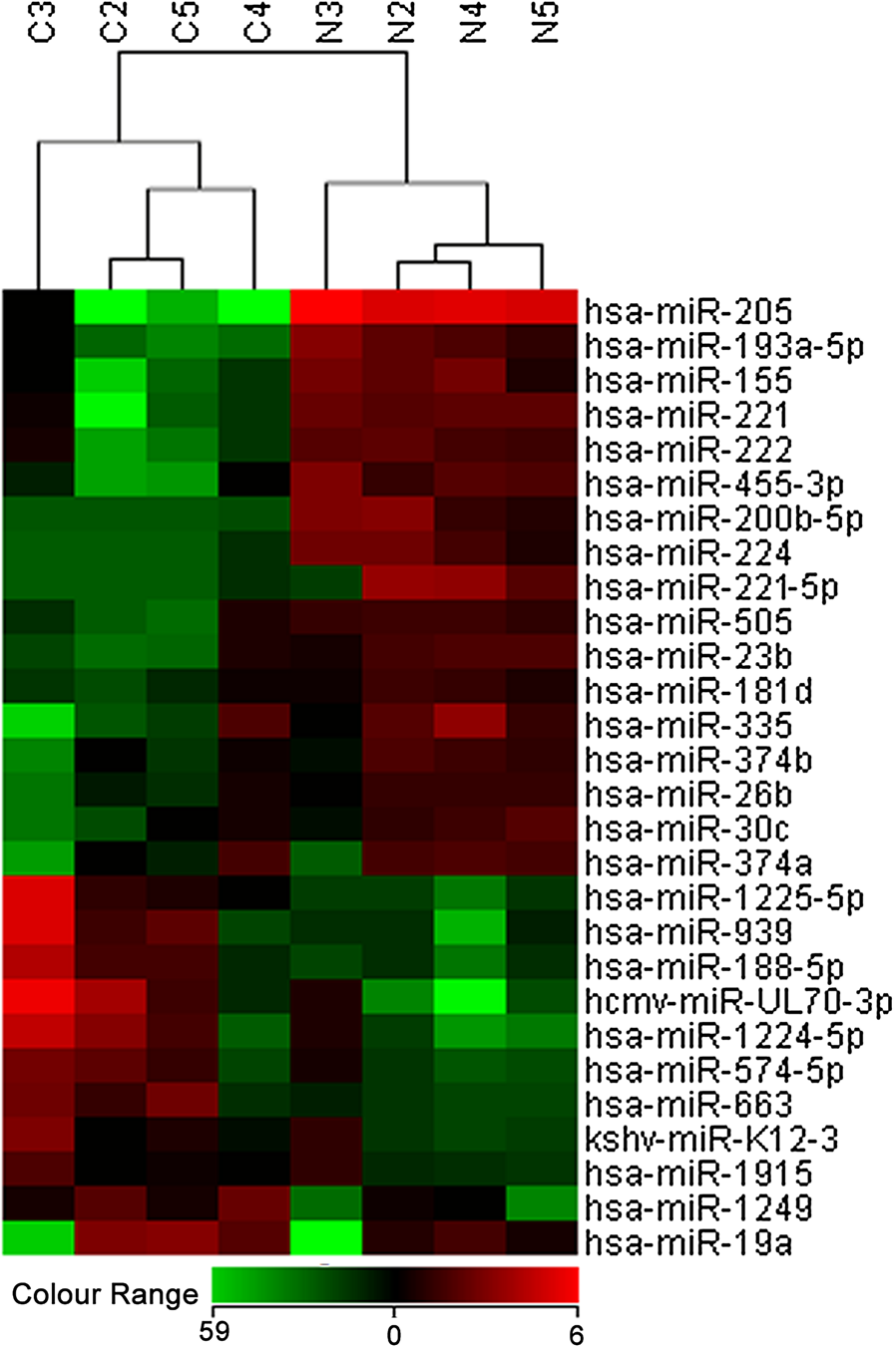
**Heat map diagram and hierarchical clustering of differentially expression miRNAs between matched tumor and adjacent benign tissue.** Each row represents a miRNA, and each column a sample either tumor tissue (C2, C3, C4, C5 and C6) or benign tissue (N2, N3, N4 and N5). The color scale shown at the bottom illustrates the relative expression level of a miRNA across all samples.

In this study, total RNA from 4 pairs of primary cancer tissue and the adjacent benign tissue of prostate gland were labelled and hybridized to miRNA microarray chip containing probes of 866 human and 89 human viral miRNAs represented. A total of 28 miRNAs (Fold time ≥1.5; *P* ≤ 0.05) were differentially expressed between tumor tissue and adjacent benign tissue of 4 prostate cancer patients. Figure 1 shows the results of the two-way hierarchical clustering of miRNAs. Each row represents a miRNA, and each column is a sample of either tumor tissues (C2, C3, C4, C5) or benign tissues (N2,N3, N4,N5). The colour scale shown at the bottom left illustrates the relative expression level of a miRNA across all samples. Of these 28 miRNAs genes, 11 miRNAs (39%) were up-regulated and 17 miRNAS (61%) were down-regulated respectively. Of the 11 miRNAs that were up-regulated in the tumor tissue, 8 miRNAs had a fold change more than two fold times. Of the 17 down-regulated miRNAs, 14 miRNAs had a fold change of more than two fold times. Additionally, miR-205 showed huge down-regulated in the tumor tissue with a fold change of greater than 50 times. Genomic locations and properties of the differentially expressed miRNAs are shown in Table 1.

**Table 1:** **The differentially expressed miRNAs in PCa (Fold change ≥ 1.5, * P *** **< 0.05)**

**Systematic name**	**miRNA microarray data**			**Chromosomal location**
	**Regulation**	**Fold change**	**P**	
hsa-miR-205	down	58.96	0.009	1:209605478-209605587[+]
hsa-miR-221	down	5.15	0.036	X:45605585-45605694[-]
hsa-miR-155	down	4.83	0.021	21:26946292-26946356[+]
hsa-miR-455-3p	down	4.39	0.014	9:116971714-116971809[+]
hsa-miR-193a-5p	down	4.31	0.001	17:29887015-29887102[+]
hsa-miR-222	down	3.94	0.036	X:45606421-45606530[-]
hsa-miR-221-5p	down	3.50	0.050	X:45605585-45605694[-]
hsa-miR-200b-5p	down	3.44	0.014	1:1102484-1102578[+]
hsa-miR-335	down	3.36	0.015	7:130135952-130136045[+]
hsa-miR-224	down	3.32	0.011	X:151127050-151127130[-]
hsa-miR-505	down	2.72	0.038	X:139006307-139006390[-]
hsa-miR-23b	down	2.70	0.033	9:97847490-97847586[+]
hsa-miR-374b	down	2.11	0.013	X:73438382-73438453[-]
hsa-miR-30c	down	2.09	0.018	1:41222956-41223044[+]
hsa-miR-26b	down	1.93	0.016	2:219267369-219267445[+]
hsa-miR-181d	down	1.70	0.031	19:13985689-13985825[+]
hsa-miR-374a	down	1.61	0.043	X:73507121-73507192[-]
hcmv-miR-UL70-3p	up	6.36	0.008	
hsa-miR-1224-5p	up	3.72	0.028	3:183959193-183959277[+]
hsa-miR-939	up	3.67	0.030	8:145619364-145619445[-]
hsa-miR-1225-5p	up	3.37	0.041	16:2140196-2140285[-]
hsa-miR-188-5p	up	3.27	0.034	X:49768109-49768194[+]
hsa-miR-1249	up	2.75	0.008	22:45596835-45596900[-]
hsa-miR-663	up	2.71	0.044	20:26188822-26188914[-]
hsa-miR-574-5p	up	2.38	0.043	4:38869653-38869748[+]
hsa-miR-19a	up	1.83	0.038	13:92003145-92003226[+]
kshv-miR-K12-3	up	1.80	0.009	
hsa-miR-1915	up	1.50	0.013	10:21785491-21785570[-]

### Validation of differentially expressed miRNA by qRT-PCR analysis

**Figure 2:**
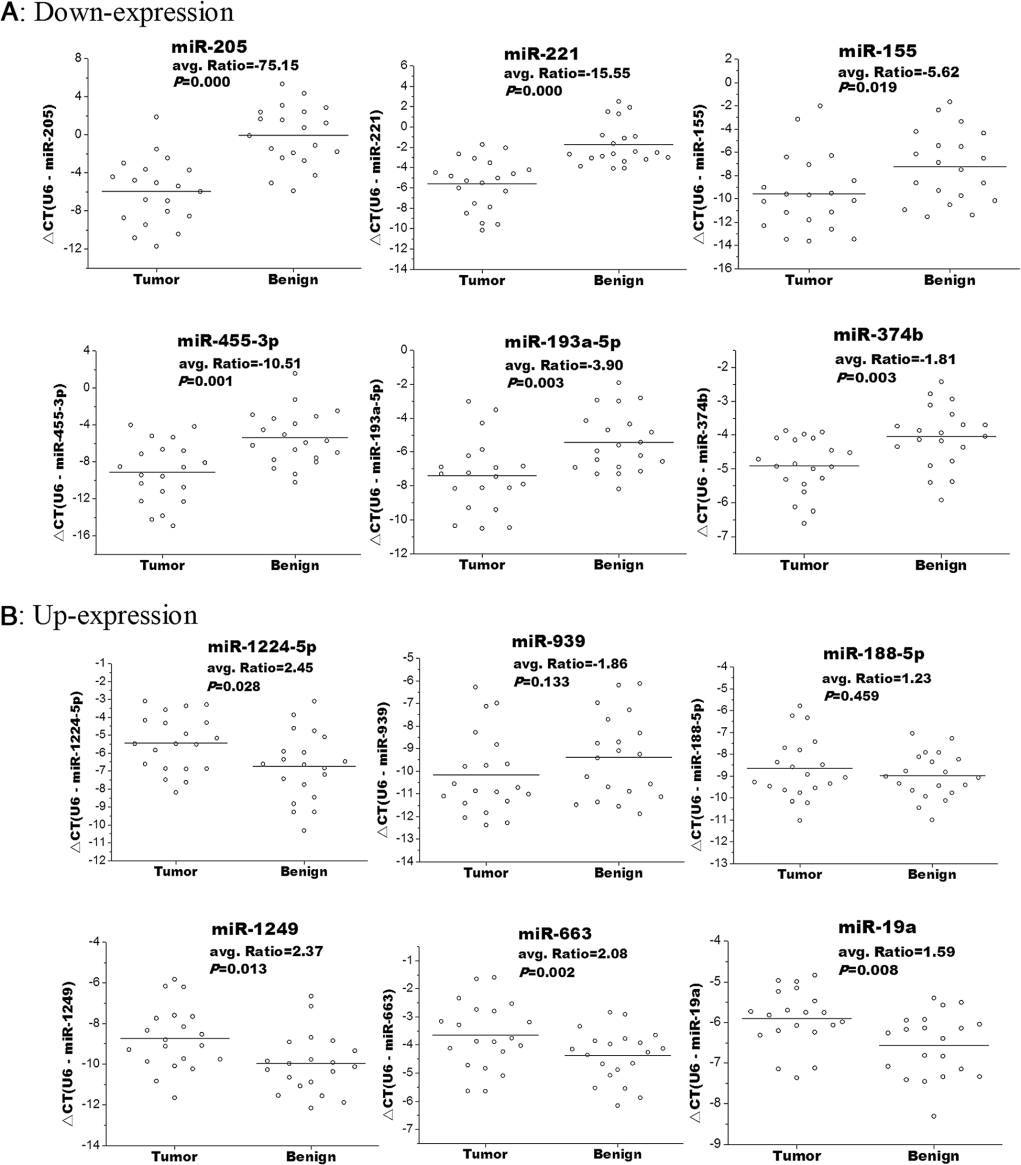
**Real-time PCR validation of microarray result.** Selected miRNAs were measured by real-time PCR in tumor tissues and paired adjacent benign tissues (n = 20). Horizontal bars represent the mean. *P* vlaues were measured by paired t-test. **(A)** real-time PCR analysis for up-regulated miRNAs in microarray result. **(B)** real-time PCR analysis for down-regulated miRNAs in microarray result.

To validate the array results in miRNA, we selected 5 down-regulated miRNAs in tumor tissue, (miR-205,miR-221, miR-155, miR-455-3P, and miR-193a-5p) and 5 up-regulated miRNAs (miR-1224-5p, miR-939, miR-188-5p, miR-1249, and miR-663) (miR-1225-5p and hcmv-miR-UL70-3p were exclude for lacking the primer for qRT-PCR) to do real-time PCR in tumor tissues and paired adjacent benign tissues (n = 20). As shown in Figure 2A, all of the 5 down-regulated miRNAs showed a significant differential expression measured by a paired t-test; in the up-regulated miRNAs group, however, although the real-time PCR results of miR-1224-5p, miR-1249, and miR-663 were consistent with the microarray data, miR-939 and miR-188-5P had no significant differential expression (*P*> 0.1).

### Functional analysis of differentially expressed miRNA by global and integrated analysis of the miRNA and mRNA expression profile

**Figure 3:**
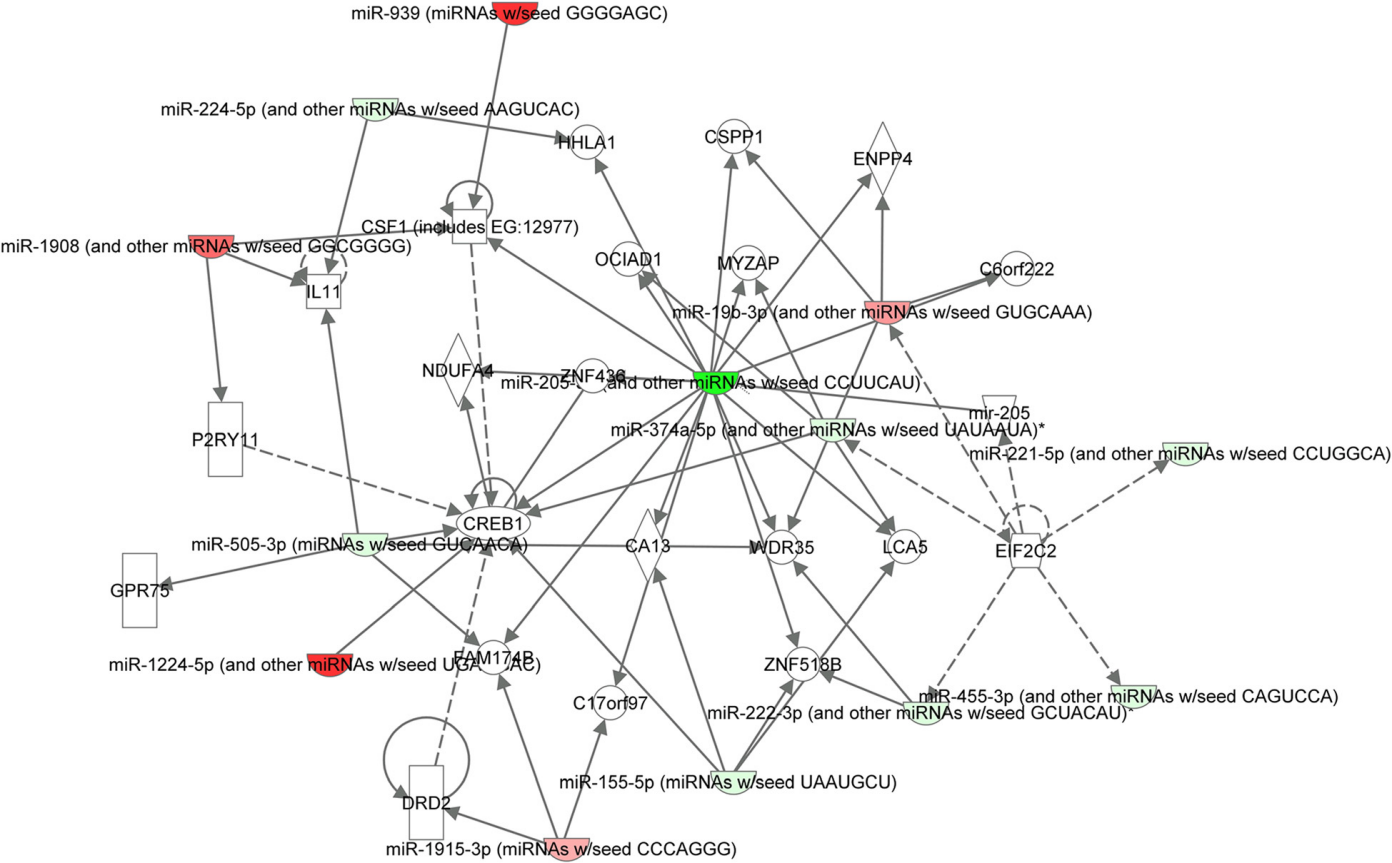
**Ingenuity pathway network analysis of differentially expressed miRNAs.** Depicting relationships among differentially expressed miRNAs in prostate cancer tissue compared with adjacent benign tissue (fold change ≥ 2, *P*< 0.05, virus miRNAs were excluded). Intensity of the red color indicates the degree of up-regulation. Intensity of the green color indicates the degree of down-regulation. Nodes are displayed using various shapes that represent the functional class of the gene product. Edges are displayed with various labels that describe the nature of relationship between the nodes: ___ represents direct relationship; ------- represents indirect relationship; →represents acts on.

**Figure 4:**
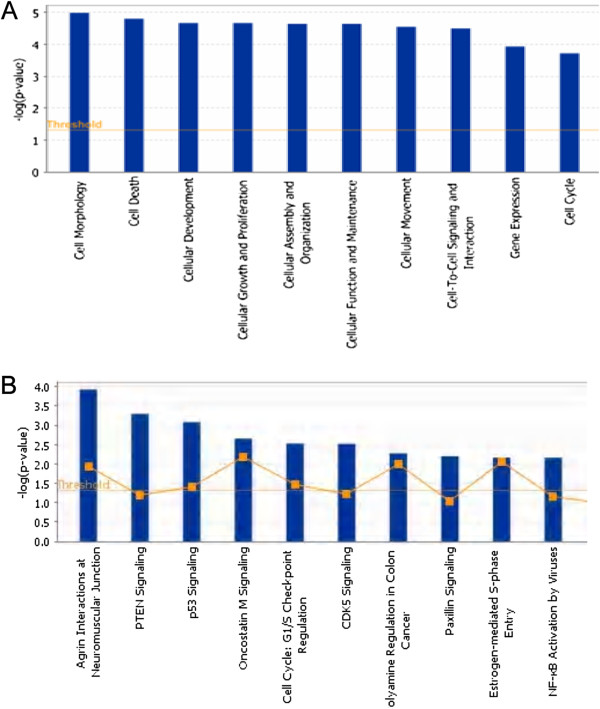
**Function and pathway analysis identified by IPA associated with targets genes.** Functional analysis for targets genes, which got from by combining the predicted target gene result by IPA software for differentially expressed miRNAs (fold change ≥ 2, *P*< 0.05) with the mRNA expression profile result (GSE NO.GSE28204, mRNA fold change ≥ 1.5, *P*< 0.05). **(A)**: The 10 most significant function directory across the entire dataset, and across multiple datasets, y-axis displays the significance. For the ratio, taller bars have more genes associated with the canonical pathway than shorter bars. **(B)** The 10 most significant canonical pathways across the entire dataset, and across multiple datasets, y-axis displays the significance. For the ratio, taller bars have more genes associated with the canonical pathway than shorter bars.

To further investigate the global association between the transcript abundance of miRNAs and their target mRNAs in prostate cancer, we used IPA pathway analysis software to perform target predication and functional analysis of the 21 differentially expressed miRNAs (fold change ≥ 2, *P*< 0.05, virus miRNAs were excluded). As shown in Figure 3, 18 differentially expressed miRNAs formed a regulated network that combined their related genes. The network was correlated with the following functions: reproductive system disease, cancer, and endocrine system disorders. We further compared the predicted targets of 21 miRNAs to the differentially expressed mRNA level data (GSE NO.GSE28204, mRNA fold change ≥ 1.5, *P*< 0.05) from the same samples. Even though there were many potential target genes predicted for the screened miRNAs, the integrated analysis mRNA and miRNA expression profile enabled us to generate an experimental target list consisting of 204 potential genes from all the predicted targets (Additional file 2: Table S2). In view of this, further analysis was restricted to genes that were potentially targeted by either up-regulated or down-regulated miRNAs. The group showing up-regulated mRNAs associated only with down-regulated miRNAs consisted of 168 mRNAs; the group showing down-regulated mRNAs associated only with up-regulated miRNAs consisted of 36 mRNAs. From this screened target set, we found that miR-155, miR-19a, miR-205a, miR-221, and miR-374b had the highest number targets (see Additional file 2: Table S2). To identify putative targets and to provide a foundation for functional analyses, we performed IPA function and pathway analysis for their targets. The screened miRNAs target genes were found to be associated with cell morphology, cell death, cellular development, cellular growth and proliferation, cellular assembly and organization, cellular function and maintenance, cellular movement, cell-to-cell signaling and interaction, gene expression, cell cycle, and so on (Figure 4A, Additional file 3: Table S3). To further elucidate the specific functions of these genes, a detailed pathway analysis was performed using ingenuity pathway analysis for all target sets (Figure 4B, Additional file 4: Table S4). The top 10 pathways were the following: Agrin Interactions at Neuromuscular Junction, PTEN Signaling, p53 Signaling, Oncostatin M Signaling, Cell Cycle: G1/S Checkpoint Regulation, CDK5 Signaling, Polyamine Regulation in Colon Cancer, Paxillin Signaling, Estrogen-Mediated S-phase Entry, and NF-κB Activation by Viruses.

### Common and specific features of miRNA expression profile in PCa tissues from Chinese patients

On the basis of these microarray experiments, we compared our data with other microarray data regarding Western PCa patients (Additional file 5: Table S5, GEO accession numbers for Taylor data, Schaefer data, and Lin data are GSE21036, GSE14857, and GSE36802, respectively). 13 differentially expressed miRNAs of our microarray data—miR-23b, miR-30c, miR-221, miR-221-5p, miR-222, miR-224, miR-205, miR-455-3p, miR-505, miR-663, miR-1224-5p, miR-1225-5p and hcmv-miR-UL70-3p—showed the same expression pattern as that of Western patients (Additional file 5: Table S5). Among these miRNAs, miR-205 was the most down-regulated miRNA both in our data and other 3 GEO data. Thus, our data reflect the shared feature of the miRNA expression profile in PCa tissues. Nevertheless, after conducting an additional literature search(Additional file 5: Table S5), we identified 15 differentially expressed miRNAs (miR-19a, miR-26b, miR-155, miR-181d, miR-188-5p, miR-193a-5p, miR-200b-5p, miR-335, miR-374a, miR-374b, miR-574-5p, miR-939, miR-1249, miR-1915, kshv-miR-K123) that have not been reported in Western PCa patients. Given the confirmed result of the qRT-PCR, miR-188-5p, miR-19a, miR-193a-5p, and miR-374b were chosen to further study the association of miRNA expression with the clinical-pathological features of PCa patients.

**Figure 5:**
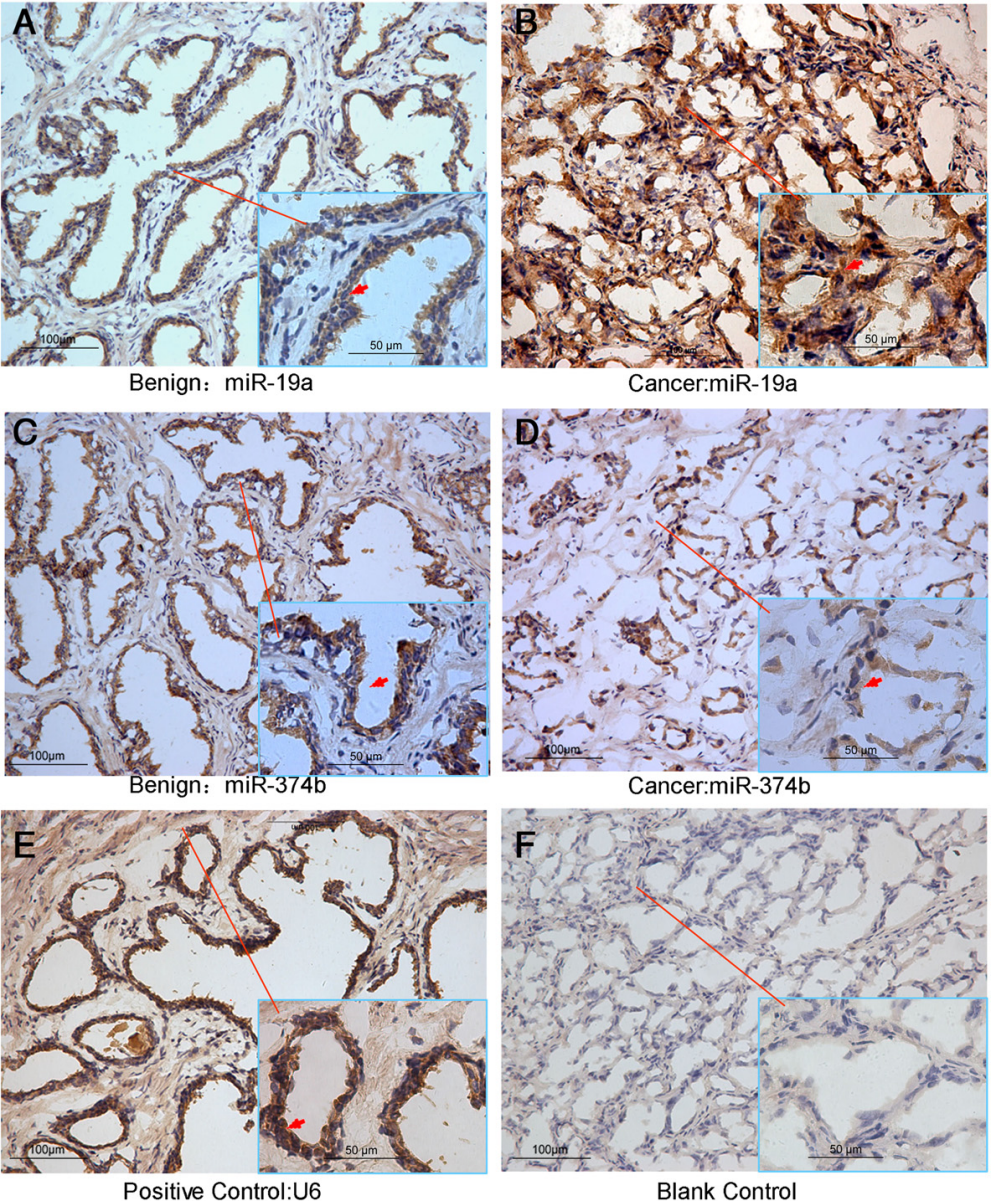
**The representative in situ hybridization images of miRNA-374b and miR-19a.** The miR-19a in-situ hybridization images in benign tissue **(A)** and cancer tissue **(B)**; The miR-394b in-situ hybridization images in benign tissue **(C)** and cancer tissue **(D)**; **(E)** is the positive control U6 RNA in-situ hybridization images and (**F)** is the blank control by replacing the RNA probe with PBS solution.

Using in situ hybridization to analyze the expression level of the chosen miRNAs in 104 PCa tissues and 25 adjacent benign prostate tissues, we found that the miR-19a showed up-regulated expression (staining score: PCa = 4.83 ± 0.76 vs. Benign = 4.15 ± 0.55, *P* = 0.004, Figure 5A–B). Conversely, the expression levels of miRNA-374b and miR-193a-5p in PCa tissues were significantly lower than those in adjacent benign prostate tissues (miRNA-374b staining score: PCa = 3.97 ± 1.17 vs. Benign = 4.70 ± 0.71, *P* = 0.032, Figure 5C–D; miR-193a-5p staining score: PCa = 3.21 ± 0.60 vs. Benign = 3.60 ± 0.55, *P* = 0.003). However, miR-188-5p showed no significant expression (data not shown, staining score: PCa = 3.49 ± 1.18 vs. Benign = 3.16 ± 0.78, *P* = 0.09).Figure 5 shows the representative images of miRNA-374b and miR-19a expression through in situ hybridization.

The clinical feature statistics revealed that only miR-374b and miR-19a had significant correlation with clinical features of prostate patients (Table 2). The reduced expression of miR-374b was frequently found in PCa tissues with a Gleason score (*P* = 0.003), pathological stage (*P* = 0.039), metastasis (*P* = 0.001), overall survival (*P* = 0.020), and PSA failure status (*P* = 0.002), whereas the up-regulation of miR-19a was only associated with high serum PSA levels (*P* = 0.020).

**Table 2:** Association of miRNA expression with the clinic-pathological features of PCa patients

**Clinical features**	**Case no.**	**miR-19a**		**miR-374b**	
			***P***		***P***
**Age (years)**					
<60	67	4.87 ± 0.62	0.43	3.81 ± 1.19	0.23
≥60	37	4.75 ± 0.97		4.08 ± 1.05	
**Serum PSA levels (ng/ml)**^**#**^					
<10	21	4.80 ± 0.66	0.020^*^	4.00 ± 0.81	0.98
≥10	82	5.16 ± 0.44		4.00 ± 1.17	
**Gleason score**^**#**^					
<8	81	4.91 ± 0.44	0.16	3.01 ± 1.571	0.003^**^
≥8	18	4.68 ± 1.11		4.28 ± 0.692	
**Clinical stage**^**#**^					
<T2A	59	4.76 ± 0.65	0.11	3.94 ± 0.146	0.21
≥T2A	41	4.96 ± 0.55		4.21 ± 0.856	
**Pathological stage**^**#**^					
T2A-T2C	65	4.82 ± 0.52	0.46	3.64 ± 1.480	0.039^*^
T3A-T4	35	4.91 ± 0.80		4.21 ± 0.775	
**Metastasis**^**#**^					
No	86	4.89 ± 0.49	0.11	4.25 ± 0.076	0.001^**^
Yes	18	4.57 ± 1.50		2.70 ± 1.661	
**Overall survival**					
Alived	94	4.91 ± 0.55	0.13	4.13 ± 0.096	0.020^*^
Dead	10	4.04 ± 1.66		2.65 ± 1.655	
**PSA failure**^**#**^					
Negative	72	4.88 ± 0.45	0.90	4.31 ± 0.081	0.002^**^
Positive	27	4.86 ± 0.90		3.36 ± 1.419	

^#^Some patients had missing data for some clinical feature.

* P < 0.05, ** P < 0.01.

**Figure 6:**
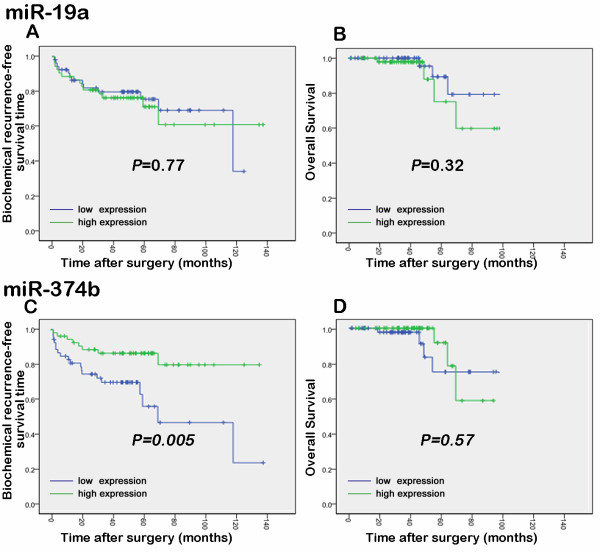
Kaplan-Meier survival curves of biochemical recurrence-free (A and C) and overall survival (B and D) for miRNA-19a and miR-374b expression in patients with prostate cancer.

The associations of the expression levels of miR-19a and miR-374b with the biochemical recurrence-free survival and the overall survival of PCa patients were analyzed using the Kaplan–Meier method (Figure 6). The data indicated that the biochemical recurrence-free survival of patients with higher miR-374b expression was significantly higher than that of those with lower miR-374b expression (*P* = 0.005). Furthermore, the multivariate analyses showed that the down-regulation of miR-374b (*P* = 0.018) and a higher Gleason score (*P* = 0.003) were both independent predictors of shorter biochemical recurrence-free survival (Table 3).

**Table 3:** Prognostic value of miRNA expression for the biochemical recurrence-free survival in univariate and multivariate analyses by Cox regression

	**Hazard ratio(95% CI)**	***P***
**Univariate**		
hsa-miR-188-5p	1.02(0.70 -1.48)	0.91
hsa-miR-19a	0.85(0.35 -1.77)	0.66
hsa-miR-193a-5p	0.60(0.35 -1.02)	0.06
hsa-miR-374b	0.61(0.50 -0.75)	0.000^**^
Gleason score	3.32(2.26-4.87)	0.000^**^
Preoperative PSA	1.00(1.00-1.01)	0.013^*^
Pathological tumor stage	3.85(1.59-9.32)	0.003^**^
Age	1.04(0.98-1.09)	0.19
Clinical stage group	0.85(0.39-1.89)	0.69
**Multivariate**		
hsamiR374b	0.38(0.17 -0.85)	0.018^*^
Gleason score	2.39(1.34-4.23)	0.003^**^
Preoperative PSA	1.01(1.00-1.01)	0.022^*^
Pathological tumor stage	2.17(0.64-7.37)	0.21

* P < 0.05, ** P < 0.01.

**Figure 7:**
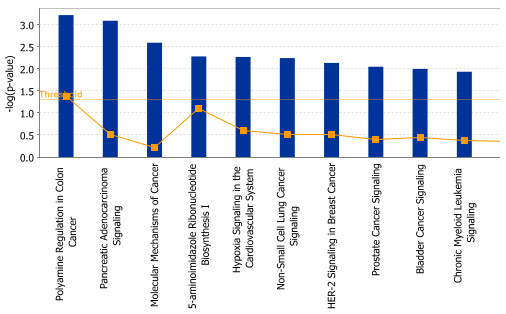
**Pathway analysis for target genes of miR-374b.** The 10 most significant canonical pathways across the entire dataset, and across multiple datasets, y-axis displays the significance. For the ratio, taller bars have more genes associated with the canonical pathway than shorter bars.

To determine whether miR-374b may modulate mRNA levels in prostate cancer patients, we compared the targets genes with our microarray expression database from our previous study [9] to determine whether any of the potential targets were significantly up-regulated in tumor tissue. We predicted that there would be decreased degradation due to loss of the miRNA targeting them. As shown in Additional file 2: Table S2, a total of 34 genes that were predicted miR-374b target genes were significantly increased in prostate cancer. IPA pathway analysis showed that some of these genes had a significant impact on the biological and clinical behavior of prostate cancer (Figure 7).

## Discussion

In this study,we elaborated on the miRNA expression profile by using microarray in 4 matched pairs of histologically confirmed tumor tissue and adjacent benign tissue. Remarkably, 28 of the miRNAs showed differential expression. Some miRNAs were subsequently validated by qRT-PCR analyses of 20 tissue pairs. Some of the miRNAs were of special interest because of their putative targets. Because putative target genes for differentially expressed miRNAs are a first step in understanding miRNAs with potential significance in the process of tumor development, we identified putative target genes for each of the differentially expressed miRNAs using IPA software. To provide a foundation for our functional analyses, we performed an integrated analysis for our previous mRNA microarray database and IPA-predicted miRNA target analysis (Additional file 2: Table S2). miR-205 has been reported to be down-regulated in prostate cancer patients and has been shown to be oncogenic [14-17]. Recently, Gandellini et al. [17] reported that miR-205 is essential for maintenance of the base membrane (BM) in prostate epithelium and pointed out that miR-205 may favour tumorigenesis by creating discontinuities in the BM. Actually, the network analysis showed centre localization of miR-205 (Figure 3), and IPA analysis for predicting targets of miR-205 showed that these targets play a role in regulating tumor morphology, which suggests that miR-205 maybe involved in prostate cancer progression. E2F1 as miR-205 target was observed in this network, interestingly, E2F1 is one of the most frequently observed potential markers for discriminating benign and malignant disease [18]. When comparing these targets shown in the network to our previous mRNA microarray data [9], however, we found that only TLK targeted by miR-205 showed significant up-regulation. Ronald et al. [19] reported that TLK expressed in a panel of PCa cell lines and their related to radioresistance. Furthermore, the expression of TLK1B in non-expressing PC-3 cells rendered them highly resistant to radiation; conversely, a knockdown to TLK1/1B in expressing DU145 reduced their radio tolerance. TLKs appear to be intimately linked to the pattern of resistance to DNA damage, and specifically to double-strand break(DSB) repair. Hence, our microarray data confirms the possibility of extending the downstream signaling of miR-205 involved in the DNA repair process.

Down-regulation of miR-221 and miR-222 has also been frequently observed in prostate cancer samples [8,20,21]. Our analysis is in concordance with these prior studies of prostate cancer. IPA analysis for targets of miR-221/222 showed that these targets play a role in regulating tumor morphology. Interestingly, some of the differentially expressed genes targeted by miR-221 and miR-222 have previously been associated with prostate cancer in Western patients, such as Ttspan13, which is overexpressed in prostate cancer and whose expression correlates with factors of favourable outcomes [22]. Among the candidates, CDON encodes the CDO protein, an orphan cell surface receptor from the immunoglobulin superfamily. Additional quantitative RT-PCR revealed that 83% of PCa tissues showed CDON overexpression. Knockdown of CDON in DU145 cells induced 5-fluorouracil-induced apoptosis and inhibited invasion ability. Therefore, CDON was suggested, as it has a high potential as a therapeutic target for PCa [23].

A down-regulated expression level of miR-23b was found in malignant tissues of prostate cancer [21]. Ectopic expression of these miRNAs significantly reduced LNCaP cancer cell growth, suggesting growth-modulatory roles for miR-23b. Majidand and his colleagues [24] reported that miR-23b expression caused a dramatic decrease in tumor growth in nude mice and attenuated Src expression. Also, the increased expression of miR-23b inhibited proliferation, colony formation, and migration/invasion and triggered G0/G1 cell cycle arrest and apoptosis in PCa [24]. As such, these findings suggest that re-expression of miR-23b may contribute to the epigenetic therapy for PCa. In our recent study [13], we also identified miR-23b as a candidate miRNA that targets PRDX3. The expression levels of miR-23b and PRDX3 in PCa tissues were found to be related to the severity of the tumor malignancy, suggesting their clinical relevance. The integrated miRNA target analysis showed that most of the targets for miR-23b are known to be involved in cellular assembly and the cell cycle. Based on the above information, we speculate that the activity of miR-23b may be conserved in Western and Chinese patients. Our integrated mRNA and miRNA analysis extends the downstream signaling of their studies and provides more comprehensive information about miR-23b in tumor growth.

In a number of studies, miRNAs such as let-7 family [25], miR-148 [26,27], and miR-210 [28,29] were also found to be up-regulated in prostate cancer, and miR-31 [30] and miR-145b [20,21,31] and miR-126 [31] were found to be down-regulated; in our study of Chinese patients, however, these miRNAs showed no significant change. The reason for these discrepancies is not clear. Recently, BoydLk et al. [32] pointed out that prostate cancer is a genetic disease characterized by multiple genomic alterations, including point mutations, microsatellite variations, and chromosomal alterations such as translocations, insertions, duplications, and deletions. The study suggests that the potential effects of the complex nature and heterogeneity of this disease might lead to miRNAs’ being differentially expressed in different populations.

Among the identified 15 differentially expressed miRNAs in Chinese patients, we selected miR-188-5p, miR-19a, miR-193a-5p, and miR-374b for further study and found that only miR-374b and miR-19a showed the association of miRNA expression with the clinical-pathological features of PCA patients.

IPA pathway analysis showed that the targets of the two miRNAs also had a critical role in cellular assembly and organization, DNA replication, recombination and repair cellular compromise, and the cell cycle. It is of interest that many of the target genes associated with the differentially expressed mRNAs in prostate cancer were found to be identical to the predicted target genes of differentially expressed miRNAs, as well as to be related to cancer and metastasis—e.g. TOP1 [33], RALA [34], SNX18, PNPT1 [35], PARD6B [36], KRAS [37] and HIPK2 [38].

Additionally, Up-regulated expression of miR-19a was found in lung cancer and colorectal cancer [39-42]. Our current study firstly reported miR-19a up-regulated in PCa with clinical-pathological features in Chinese patients but not in non-Chinese patients. The result provides a starting point for future research into the function of miR-19a and suggests that miR-19a up-regulated may involve heterogeneous disorder of Chinese patients.

MiR-374b was reported as being involved in multiple physical functions and diseases, such as adipocyte differentiation [43,44], lipid metabolism, renal Ca^2+^ homeostasis [45], vascular permeability in injury [46], and lung cancer [47]. Our results showed that miR-374b is an independent predictor of shorter biochemical recurrence-free survival of PCa by in situ hybridization. Interestingly, it was recently reported that miR-374 in seminal plasma can, as a diagnostic biomarker for male infertility, combine with other miRNAs [48]. As such, our findings suggest that the further study of the miR-374b expression level in prostate fluid or serum of prostate cancer patients may promote the clinical application of the miR-374b as PCa biomarker.

## Conclusions

Our study supports the concept that alternations of miRNA expression play an important role in prostate carcinogenesis. miR-23b, miR-220, miR-221, miR-222 and miR-205 maybe common critical therapeutic targets in the different populations. However, while a miRNA potentially has hundreds of target genes, relatively few targets have been experimentally validated. Our integrated analysis for mRNA microarray and miRNA microarray will help us to investigate the effect of specifically inhibiting/enhancing the function of miRNAs on the activity of their putative target genes, gene translation and the various stages of cancer development. This study also identifies those 15 specific expressed miRNAs in Chinese patients. The clinical feature statistics reveal that only miR-374b and miR-19a have significant correlation with clinic-pathological features, which suggest miR-374b and miR-19a may be involved in prostate cancer progression of Chinese patients.

## Competing interests

The authors declare that they have no competing interests.

## Authors’ contributions

HH and ZH: participated in the study analysis and interpretation of data, performed most of the experiments and help to draft the manuscript. QD, XL, XF, ZL, YD, GQ and CC: participated in clinical sample collection and clinical data analysis, helped to do some experiments and statistical analysis. JC and FJ: participated in the array data processing, analysis and performed statistical analysis, and help to draft the tables and figures. XL: participated in the concept origination, design, analysis and interpretation of data, drafting all of figures and manuscript. WZ: participated in the concept origination, design and coordination, acquisition of data, analysis and interpretation of data, material support for obtained funding, and supervised study. All authors read and approved the final manuscript.

## Supplementary Material

Additional file 1: Table S1 Clinical features of all patients. Click here for file

Additional file 2: Table S2 The integrated analysis for miRNA microarray and mRNA microarray. Click here for file

Additional file 3: Table S3 Functions analysis of miRNAs target genes. Click here for file

Additional file 4: Table S4 Pathway analysis of miRNAs target genes. Click here for file

Additional file 5: Table S5 Comparison diffferent expressed miRNAs in Chinese Pca patients with non-Chinese Pca patients. Click here for file
