# Supplementary material for: Global analysis of the differentially expressed miRNAs of prostate cancer in Chinese patients
Source: BMC Genomics. 2013 Nov 5;14:757. doi: 10.1186/1471-2164-14-757 (PMC4008360; doi:10.1186/1471-2164-14-757)
Supplement: Additional file 1: Table S1 — Clinical features of all patients. [file 1471-2164-14-757-S1.pdf]

Table S1 Clinical features of all patients

Sample Type & Clinical Features	Experiment Type (cases)		
	Microarray	QRT-PCR	ISH
**Prostate Cancer**	5	20	104
**Mean age (range, years)**	69.2±10.8 (54-80)	73.6±11.5 (54-85)	59.80 ± 7.07 (43-86)
< 60	0	4	67
≥60	4	16	37
**Serum PSA levels(ng/ml)**			#
<10	1	5	21
⩾10	3	15	82
**Gleason Score**			#
<8	2	12	81
≥8	2	7	18
**Clinical Stage**			#
<T2A	0	13	59
≥T2A	4	6	41
**Metastasis**	0	4	18#
**Adjacent Benign Prostate Tissue**	5	20	25

Some patients had missing data for some clinical feature.
