# Supplementary material for: Global analysis of the differentially expressed miRNAs of prostate cancer in Chinese patients
Source: BMC Genomics. 2013 Nov 5;14:757. doi: 10.1186/1471-2164-14-757 (PMC4008360; doi:10.1186/1471-2164-14-757)
Supplement: Additional file 5: Table S5 — Comparison diffferent expressed miRNAs in Chinese Pca patients with non-Chinese Pca patients. [file 1471-2164-14-757-S5.pdf]

Table S5. Comparison differentially expressed miRNAs in Chinese Pca patients with non-Chinese Pca patients

Systematic Name	Our data	Taylor data	Schaefer data	Lin Data
hsa-miR-205	down	down	down	down
hsa-miR-221	down	down	down	down
hsa-miR-155	down	-	-	-
hsa-miR-455-3p	down	down	-	down
hsa-miR-193a-5p	down	-	-	-
hsa-miR-222	down	down	down	down
hsa-miR-221-5p	down	down	-	-
hsa-miR-200b-5p	down	-	-	-
hsa-miR-335	down	-	-	-
hsa-miR-224	down	down	-	-
hsa-miR-505	down	down	-	-
hsa-miR-23b	down	down	-	-
hsa-miR-374b	down	-	-	-
hsa-miR-30c	down	down	-	-
hsa-miR-26b	down	-	-	-
hsa-miR-181d	down	-	-	-
hsa-miR-374a	down	-	-	-
hcmv-miR-UL70-3p	up	up	-	-
hsa-miR-1224-5p	up	-	-	up
hsa-miR-939	up	-	-	-
hsa-miR-1225-5p	up	up	-	-
hsa-miR-188-5p	up	-	-	-
hsa-miR-1249	up	-	-	-
hsa-miR-663	up	up	-	-
hsa-miR-574-5p	up	-	-	-
hsa-miR-19a	up	-	-	-
kshv-miR-K12-3	up	-	-	-
hsa-miR-1915	up	-	-	-
